# Cariprazine Augmentation in Treatment-Resistant Bipolar Depression: Data from a Retrospective Observational Study

**DOI:** 10.2174/1570159X22666240129095852

**Published:** 2024-01-29

**Authors:** Elena Teobaldi, Enrico Pessina, Azzurra Martini, Carlo Ignazio Cattaneo, Domenico De Berardis, Vassilis Martiadis, Giuseppe Maina, Gianluca Rosso

**Affiliations:** 1 Department of Neurosciences, University of Turin, Turin 10126, Italy;; 2 Department of Mental Health, Community Mental Health Center, ASL Cuneo 2, Alba, Italy;; 3 Department of Mental Health, ASL Novara, Novara, Italy;; 4 Department of Mental Health, Psychiatric Service for Diagnosis and Treatment, Hospital “G. Mazzini”, ASL 4, Teramo, Italy;; 5 Department of Mental Health, ASL Napoli 1 Centro, Napoli, Italy;; 6 Department of Neurosciences, San Luigi Gonzaga University Hospital, Orbassano, Turin, Italy

**Keywords:** Bipolar disorder, bipolar depression, treatment-resistance, TRBD, cariprazine, hamilton depression rating scale

## Abstract

**Background:**

Treatment-resistant bipolar depression is one of the leading problems in psychiatry with serious consequences on patients functioning, quality of life and resource utilization. Despite this, there is a lack of consensus on diagnostic criteria and treatment algorithms.

**Objective:**

The objective of the present study is to assess the acute effectiveness and tolerability of cariprazine in the management of treatment resistant bipolar depression.

**Methods:**

This is a four weeks retrospective multicentric observational study on patients with treatment resistant bipolar depression receiving cariprazine in augmentation to the current treatment. Cariprazine dosage changed during the follow-up period according to clinical judgment. Since data followed a non-normal distribution, non-parametric tests were used to pursue the analysis. The effectiveness of cariprazine was assessed through the mean change in Hamilton Depression rating scale (HAM-D) scores from baselin

**Results:**

Fifty-one patients were enrolled. Four patients (7.8%) discontinued cariprazine mainly due to adverse events. Mean cariprazine dose was 1.7 mg/day. The mean HAM-D score decreased significantly from baseline (T0) to week 4 (T4) at each evaluation point. Fourty-five one percent of the patients benefited of cariprazine add-on strategy: 23.5% achieved a clinical response and 21.6% were remitters. Among the completers, 70.6% experienced at least one adverse event. All side effects were mild to moderate.

**Conclusion:**

Cariprazine seems to be an effective and well tolerated option in the management of patients with treatment resistant bipolar depression.

## 1 INTRODUCTION

Depressive episodes represent a major issue in the management and treatment of bipolar disorder (BD). They account for most of the time spent in illness [1-4] and contribute to the high burden of disability and reduced quality of life, as well as the increased risk of suicide that characterize the disorder [5-7]. Despite the high prevalence and burden of morbidity, there are limited licensed medications for bipolar depression [8, 9], and only a minority of patients achieve remission after adequate treatment. Non-response occurred in 40% of patients after eight weeks of quetiapine [10], and the rate is even higher with other first-line medications such as lithium, lamotrigine or fluoxetine-olanzapine combination [10-13].

Non-pharmacological approaches, such as transcranial direct current stimulation (tDCS) or repetitive transcranial magnetic stimulation (rTMS), have also been proposed for the treatment of bipolar depression [14, 15], with considerable heterogeneity in response rate.

Moreover, the definition of treatment-resistant bipolar depression (TRBD) remains a subject of ongoing debate and lacks a shared operative definition [16]. Among the proposed models, Hidalgo-Mazzei and colleagues [9] defined TRBD as “patient failed to reach sustained symptomatic remission at least for 8 consecutive weeks or did not tolerate two different trials at adequate therapeutic doses during 8 weeks either with at least two treatments in monotherapy or at least one treatment monotherapy and one treatment in combination”. However, these criteria were difficult to be applied worldwide because many of the drugs listed are not licensed in every country for the treatment of bipolar depression (*e.g*., lurasidone). Conversely, some licensed drugs are missing.

Cariprazine is an atypical antipsychotic, approved in Europe for the treatment of schizophrenia, that acts as a partial agonist at dopamine D2/D3 (with a higher affinity for D3) and serotonin 5HT1A receptors and as a weak antagonist of 5HT2A receptors [17]. Affinity at both D2 and D3 receptors is believed to be involved in strong antipsychotics efficacy as well as treatment advantages such as antidepressant, antianhedonic and pro-cognitive effects; moreover, Partial antagonism at the 5-HT1A receptors may contribute to its antidepressant effects [18, 19]. Initially licensed by the FDA for the treatment of schizophrenia (1,5-6 mg/day) and acute manic/mixed episodes (3-6 mg/day), cariprazine was subsequently approved for the treatment of bipolar depression (1,5-3 mg/day) and as an adjunctive treatment for unipolar major depressive episodes (1,5-3 mg/day), mainly due to its aforementioned pharmacodynamics properties.

Despite the evidence of efficacy and safety in the treatment of bipolar depression [20], to the best of our knowledge, there is a lack of data on the efficacy of cariprazine in TRBD. Therefore, the aim of this retrospective observational study was to assess the effectiveness and safety of cariprazine in real-world patients with treatment-resistant bipolar depression.

## 2 MATERIALS AND METHODS

### 2.1 Study Design and Patients

Data was derived from an independent retrospective multicentric observational study aimed at analyzing the acute effectiveness and tolerability of cariprazine in the management of treatment-resistant bipolar depression.

Clinical records of inpatients and outpatients with a diagnosis of bipolar disorder (DSM-5) consecutively admitted or referred to the Psychiatric Unit of San Luigi Gonzaga University Hospital in Orbassano (University of Turin, Italy), the Mental Health Department of Alba and Bra (Cuneo, Italy), the Department of Mental Health of “G. Mazzini” Hospital (Teramo, Italy), the Department of Mental Health of Borgomanero (Novara, Italy) and the Department of Mental Health of Napoli 1 (Napoli, Italy) from June 2022 to February 2023, were analyzed.

The inclusion criteria were the following: a) Age of majority; b) Principal diagnosis of bipolar disorder according to DSM-5 criteria; c) Current major depressive episodes; d) Being treatment resistant according to the operational definition proposed by Murphy and colleagues [21] and already used for studies on TRBD [22] “Tried and failed at least two adequate trials (in dosage achieved and duration) from two classes of antidepressants and two classes of mood stabilizers (including atypical antipsychotic agents)”; e) Being currently treated with at least one mood stabilizers (lithium or valproate) in therapeutic range; f) Receiving cariprazine in augmentation to the current treatment.

All subjects referred to our services did sign a written informed consent to have their clinical data potentially used for teaching or research purposes anonymously treated. A specific request was made to the local Ethical Committee in order to have access to the clinical records of all BD patients who agreed and signed the abovementioned written informed consent.

Cariprazine starting dose and dosage changes were established according to clinical judgment.

### 2.2 Assessment

Socio-demographic, clinical and safety information were collected for each subject from medical reports. Patients underwent control visits according to clinical practice. Clinical symptoms were assessed by means of the Hamilton Depression rating scale (HAM-D), Hamilton Anxiety rating scale (HAM-A), Young Mania Rating Scale (YMRS) and Bipolar depression rating scale (BDRS). For the purpose of this study, medical records were analyzed at the start of treatment with cariprazine and then weekly for four weeks.

All psychiatric diagnoses and clinical assessments were made by a psychiatrist with several years of experience.

The effectiveness of cariprazine was assessed, evaluating the mean change of HAM-D scores from baseline to endpoint. Moreover, qualitative analysis was performed, calculating rates of responders (HAM-D scores mean reduction of at least 50%) and remitters (HAM-D scores < 7).

### 2.3 Statistical Analysis

Sociodemographic and clinical data were presented as mean ± standard deviation for quantitative variables and in percentage (%) for categorical variables. Given a significance level of 0.05, the post hoc power analysis on the sample size of 51 patients showed a statistical power > 95% related to HAM-D mean scores.

Since data followed a non-normal distribution at baseline (Kolmogorov-Smirnov test: 0.139; *p*: 0.023), non-parametric tests were used. The Wilcoxon test was used to assess the changes in the clinical scales during the observational period. For missing values, a “Last Observation Carried Forward” (LOCF) approach was applied. In addition, patients were categorized into responders/non-responders groups according to the reduction in HAM-D scores and were compared with Kruskal-Wallis non-parametric test for continuous variables and with chi-square (χ_2_) for categorical variables. Significance was set at *p <* 0.05. All analyses were performed using IBM SPSS 28.0 software (Armonk, NY, USA).

## 3 RESULTS

Fifty-one patients fulfilled the inclusion criteria and were enrolled. Among these, 30 patients (58.8%) were female. The mean age of the sample was 44.08 ± 12.64 years. Thirty-four patients (66.7%) suffered from bipolar disorder type II, while 17 (33.3%) were from BD type I; the mean age at onset was 25.45 ± 6.98 years. Two-thirds of patients (66.7%) had other comorbid psychiatric disorders. All patients were on mood stabilizers (76.5% were treated with lithium and the remaining 23.5% with valproate), and they were treated with at least one antidepressant of different classes. The mean HAM-D score at baseline was 24.86 ± 4.9. All socio-demographic and clinical characteristics at baseline are shown in Table **1**. The mean cariprazine dosage prescribed during the follow-up period was 1.7 mg/day.

**Fig. (1):**
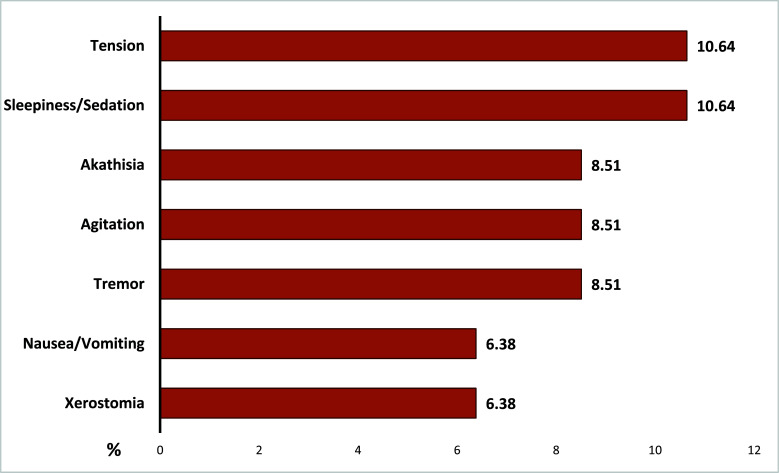
Most common adverse events in completers (N: 47). Adverse events <5%: insomnia, constipation, headache, rigidity, weight gain.

Forty-seven patients (92.2%) completed the observation period, and four patients (7.8%) dropped out. At week one, two subjects dropped out due to adverse events (AES: agitation and akathisia, respectively), while at week two, one subject discontinued cariprazine due to severe agitation and one subject was lost at follow-up. All dropouts occurred within week two. Among the completers, 36 patients (70.6%) experienced at least one AEs (Fig. **1**).

**Fig. (2):**
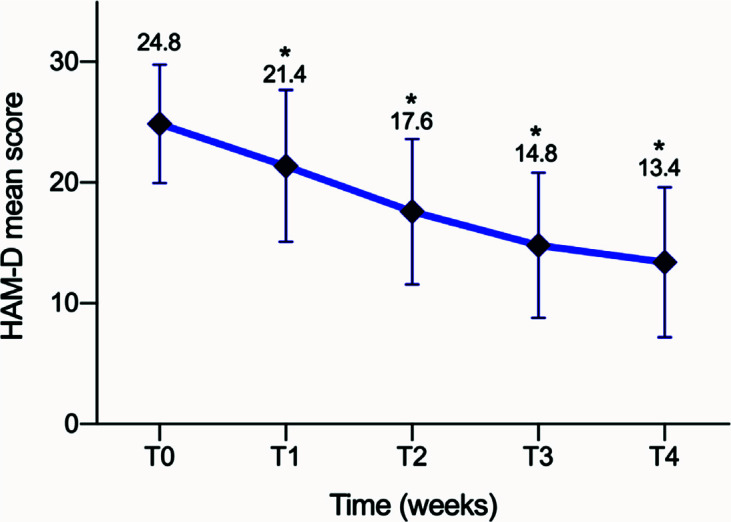
Mean reduction of Hamilton Depression rating scales (HAM-D) scores during follow-up. *p < 0.05.

The non-parametric Wilcoxon test showed an overall significant reduction in HAM-D scores and BDRS scores at all time-point compared to the baseline. The mean HAM-D score decreased significantly from baseline (T0) to week 4 (T4) at each evaluation point: 24.86 ± 4.9 at T0, 21.37 ± 6.3 at T1 (Z: -4.96; *p <* .001), 17.59 ± 6.0 at T2 (Z: -5.32; *p <* .001), 14.81 ± 6.0 at T3 (Z: -5.855; *p <* .001) and 13.40 ± 6.2 at T4 (Z: -5.87; *p <* .001) (Fig. **2**). The same applies for BDRS score: 32.08 ± 3.9 at baseline (T0), 28.20 ± 6.5 at T1 (Z: -4.773, *p <* .001), 23.22 ± 7.6 at T2 (Z:-5.617, *p <* .001), 19.26 ± 8.4 at T3 (Z: -5.844, *p <* .001) and 17.28 ± 9.47 at T4 (Z: -5.902, *p <* .001).

At the end of the observation period, of 51 patients, 12 patients (23.5%) achieved a clinical response (HAM-D reduction ≥ 50%), while 11 patients (21.6%) were remitters (HAM-D scores < 7).

The mean HAM-A score was 26.63 ± 7.4 at baseline (T0), 24.06 ± 6.9 at T1 (Z: -3.416, *p <* .001), 20.37 ± 7.6 at T2 (Z: -4.298, *p <* .001), 17.19 ± 7.7 at T3 (Z: -4.777, *p <* .001), 15.66 ± 7.9 at T4 (Z:-4.976, *p <* .001). As for the YMRS mean scores, no significant differences from baseline were found at any time point, except for a slight but significant increase at T1 (YMRS at baseline: 1.29 ± 1.5, at T1: 1.61 ± 1.6; Z: - 2.182, *p*: 0.029). The LOCF analysis confirmed all the results mentioned above.

Comparing baseline characteristics of responders (n:23, 45.1%) and non-responders (n:28, 54.9%), no differences in depressive symptoms at baseline were found, with both HAM-D and BDRS (*p*: 0.615 and *p*: 0.754, respectively). The Mann-Whitney U test showed higher scores of the HAM-A at baseline in the responder's group (responders: 28.91 ± 7.83, non-responders: 24.75 ± 6.60, Z: -1.88, *p*: 0.059). Lastly, no differences were found between the two groups, neither examining the type of bipolar disorders (χ2: 1.940, *p*: 0.164) nor considering the mood stabilizer treatment at baseline (χ_2_:0.075, *p*: 0.785).

## 4 DISCUSSION

To our knowledge, this is the first study focusing on the effectiveness and tolerability of cariprazine in TRBD real-world patients as an add-on strategy.

Treatment-resistant depression is one of the leading problems in psychiatry with serious consequences in terms of patient's functioning and quality of life as well as resource utilization in both Major Depressive Disorder and BD [16, 23-25]. Despite this, there is a lack of consensus on diagnostic criteria and treatment algorithms for both disorders [23, 26, 27], thus also preventing the estimation of the true prevalence of the condition.

Although not licensed in all countries, cariprazine is one of the so-called third-generation antipsychotics that has shown evidence of efficacy and safety in the treatment of bipolar depression [4], to be included as first-line treatments in guidelines [28]. Nevertheless, in Europe, cariprazine can only be used off-label for bipolar depression in clinical practice.

The mean cariprazine dosage (1.7 mg/day) used in our sample was in line with doses used in non-treatment-resistant bipolar depression [20], highlighting how higher dosages are not usually prescribed in bipolar depression, both treatment and non-treatment-resistant.

Our results show that cariprazine can reduce depressive and anxious symptoms in real-world TRBD patients in the short term period. Indeed, at the end of a 4-week observation period, 23.5% of patients met the criteria for a clinical response, and another 21.6% achieved clinical remission. As a result, an average of 45% of patients derived benefits from the cariprazine add-on strategy. A post-hoc analysis of three randomized placebo-controlled studies on the efficacy of cariprazine in (non-resistant) bipolar depression has shown only slightly higher remission rates, 30% and 22.4% for the 1.5 mg/day and 3 mg/day, respectively [4]. This difference, although small, can be explained by the fact that real-world patients are more complex than those enrolled in RCTs [29]. On the other hand, it could be argued that cariprazine might be effective both in TRBD patients and non-TRDB patients, with efficacy results depending more on other characteristics of the patients, such as symptom dimensions. Indeed, its receptor profile and initial clinical findings provide the basis for believing that cariprazine plays a role in the regulation of motivation and reward-related behavior and may be able to offer improvement in dimensions such as anhedonia and cognition [18, 30-33]. In our sample, the type of bipolar disorder and the patient’s psychopharmacological treatments at baseline did not seem to influence the response to therapy.

Concerning tolerability, we found a high rate of AEs (70.6%), especially restlessness/tension, sleepiness, akathisia and tremors, consistent with those most frequently reported in clinical trials with capiprazine in treating bipolar depression [34]. Although the side effects were mild to moderate, three out of four patients discontinued the study because of the occurrence of AEs (restlessness and akathisia). In a pooled post-hoc analysis on the safety of cariprazine in bipolar depression, the percentage of patients experiencing any AE was lower (59.5%) [35]. However, the higher prevalence of AEs emerged in our real-world sample could be mainly explained by the fact that the strict inclusion and exclusion criteria used in RCTs may exclude most patients encountered in clinical practice [29].

The aforementioned results should be interpreted in light of several limitations of the study, including the retrospective observational design of the study and its very short duration (four weeks). Furthermore, the study lacks a control group of healthy subjects or an active comparator. We acknowledge that the sample size is small, which prevented us from conducting sub-analyses on specific patient characteristics. Furthermore, patients with a high rate of psychiatric comorbidities were included, which may have compromised the study results, such as affecting treatment adherence. However, the comorbidity rate is high in patients with BD, making the study sample representative of real-world TRBD patients. In addition, any dose adjustments required by the concomitant administration of drugs that alter the enzymatic metabolism of cariprazine have not been evaluated. Lastly, tolerability was assessed by reviewing the medical records without reconstructing more specific scales for assessing side effects.

## CONCLUSION

Despite all the limitations, this is the first study evaluating the effectiveness and safety of cariprazine in the treatment of patients with treatment-resistance bipolar depression. Given the high prevalence of these conditions and their impact on quality of life and functioning, studies focusing on the pharmacological management of these specific groups of patients in the real world are sorely needed.

## Figures and Tables

**Table 1:** Baseline demographic and clinical characteristics of the sample.

**Parameters**	***N* = 51**
**Age,** years (mean ± SD)	44.1 ± 12.6
**Sex,** *n* (%) Male Female	21 (41.2) 30 (58.8)
**Marital Status,** *n* (%): Single Married Divorced Widowed	13 (25.5) 31 (60.8) 5 (9.8) 2 (3.9)
**Educational Level,** years (mean ± SD)	13.2 ± 3.8
**Working for Pay,** *n* (%) Yes No	27 (52.9) 24 (47.1)
**Age at Onset of Bipolar Disorder,** years (mean ± SD)	25.5 ± 6.9
**Bipolar Disorder Subtype,** *n* (%) I II	17 (33.3) 34 (66.7)
**Suicide Attempts Lifetime,** *n* (%) Yes No	26 (51.0) 25 (49.9)
**Psychiatric Comorbidities,** *n* (%) Yes No	34 (66.7) 17 (33.3)
**Type of Psychiatric Comorbidities,** *n* (%) OCD Anxiety disorders SUD Eating disorders	9 (17.7) 20 (39.2) 4 (7.8) 1 (2.0)
**Mood Stabilizer,** *n* (%) Lithium Valproate	39 (76.5) 12 (23.5)
**Class of Antidepressant,** *n* (%) SSRI SNRI Multimodal TCA	28 (61.0) 6 (13.0) 6 (13.0) 6 (13.0)
**HAM-D Scores,** (mean ± SD)	24.9 ± 4.9
**BDRS Scores,** (mean ± SD)	32.1 ± 3.9
**HAM-A Scores,** (mean ± SD)	26.6 ± 7.4
**YMRS Scores,** (mean ± SD)	1.29 ± 1.5

**Abbreviations:** OCD: Obsessive-Compulsive Disorder; SUD: Substance Use Disorder; HAM-D: Hamilton Depression rating scales; HAM-A: Hamilton anxiety rating scale; YMRS: Young Mania rating scale; BDRS: Bipolar Depression rating scale; SSRI: Selective serotonin reuptake inhibitor; SNRI: Serotonin and Norepinephrine Reuptake Inhibitor; TCA: tricyclic antidepressant.

## Data Availability

Not applicable.

## LIST OF ABBREVIATIONS

BD: Bipolar Disorder
BDRS: Bipolar Depression Rating Scale
rTMS: Repetitive Transcranial Magnetic Stimulation
tDCS: Transcranial Direct Current Stimulation
TRBD: Treatment-resistant Bipolar Depression

## STANDARDS OF REPORTING

STROBE guidelines and methodology were followed.

## References

[1] Judd L.L., Akiskal H.S., Schettler P.J., Endicott J., Maser J., Solomon D.A., Leon A.C., Rice J.A., Keller M.B. (2002). The long-term natural history of the weekly symptomatic status of bipolar I disorder.. Arch. Gen. Psychiatry.

[2] Judd L.L., Akiskal H.S., Schettler P.J., Coryell W., Endicott J., Maser J.D., Solomon D.A., Leon A.C., Keller M.B. (2003). A prospective investigation of the natural history of the long-term weekly symptomatic status of bipolar II disorder.. Arch. Gen. Psychiatry.

[3] Colom F., Vieta E., Daban C., Pacchiarotti I., Sánchez-Moreno J. (2006). Clinical and therapeutic implications of predominant polarity in bipolar disorder.. J. Affect. Disord..

[4] McIntyre R.S., Suppes T., Earley W., Patel M., Stahl S.M. (2020). Cariprazine efficacy in bipolar I depression with and without concurrent manic symptoms: Post hoc analysis of 3 randomized, placebo-controlled studies.. CNS Spectr..

[5] Kupka R.W., Altshuler L.L., Nolen W.A., Suppes T., Luckenbaugh D.A., Leverich G.S., Frye M.A., Keck P.E., McElroy S.L., Grunze H., Post R.M. (2007). Three times more days depressed than manic or hypomanic in both bipolar I and bipolar II disorder.. Bipolar Disord..

[6] Malhi G.S., Ivanovski B., Hadzi-Pavlovic D., Mitchell P.B., Vieta E., Sachdev P. (2007). Neuropsychological deficits and functional impairment in bipolar depression, hypomania and euthymia.. Bipolar Disord..

[7] Gutirrez-Rojas L., Gurpegui M., Ayuso-Mateos J.L., Gutirrez-Ariza J.A., Ruiz-Veguilla M., Jurado D. (2008). Quality of life in bipolar disorder patients: A comparison with a general population sample.. Bipolar Disord..

[8] Bauer M., Ritter P., Grunze H., Pfennig A. (2012). Treatment options for acute depression in bipolar disorder.. Bipolar Disord..

[9] Hidalgo-Mazzei D., Berk M., Cipriani A., Cleare A.J., Florio A.D., Dietch D., Geddes J.R., Goodwin G.M., Grunze H., Hayes J.F., Jones I., Kasper S., Macritchie K., McAllister-Williams R.H., Morriss R., Nayrouz S., Pappa S., Soares J.C., Smith D.J., Suppes T., Talbot P., Vieta E., Watson S., Yatham L.N., Young A.H., Stokes P.R.A. (2019). Treatment-resistant and multi-therapy-resistant criteria for bipolar depression: Consensus definition.. Br. J. Psychiatry.

[10] De Fruyt J., Deschepper E., Audenaert K., Constant E., Floris M., Pitchot W., Sienaert P., Souery D., Claes S. (2012). Second generation antipsychotics in the treatment of bipolar depression: A systematic review and meta-analysis.. J. Psychopharmacol..

[11] Geddes J.R., Calabrese J.R., Goodwin G.M. (2009). Lamotrigine for treatment of bipolar depression: Independent meta-analysis and meta-regression of individual patient data from five randomised trials.. Br. J. Psychiatry.

[12] Yatham L.N., Calabrese J.R., Kusumakar V. (2003). Bipolar depression: Criteria for treatment selection, definition of refractoriness, and treatment options.. Bipolar Disord..

[13] Sidor M.M., MacQueen G.M. (2011). Antidepressants for the acute treatment of bipolar depression: A systematic review and meta-analysis.. J. Clin. Psychiatry.

[14] D’Urso G., Toscano E., Barone A., Palermo M., Dell’Osso B., Di Lorenzo G., Mantovani A., Martinotti G., Fornaro M., Iasevoli F., de Bartolomeis A. (2023). Transcranial direct current stimulation for bipolar depression: systematic reviews of clinical evidence and biological underpinnings.. Prog. Neuropsychopharmacol. Biol. Psychiatry.

[15] Kishi T., Ikuta T., Sakuma K., Hatano M., Matsuda Y., Kito S., Iwata N. (2023). Repetitive transcranial magnetic stimulation for bipolar depression: A systematic review and pairwise and network meta-analysis.. Mol. Psychiatry.

[16] Fornaro M., De Berardis D., Koshy A., Perna G., Vancampfort D., Stubbs B., Valchera A. (2016). Prevalence and clinical features associated with bipolar disorder polypharmacy: A systematic review.. Neuropsychiatr. Dis. Treat..

[17] Stahl S.M. (2016). Mechanism of action of cariprazine.. CNS Spectr..

[18] Gyertyán I., Sághy K., Laszy J., Elekes O., Kedves R., Gémesi L.I., Pásztor G., Zájer-Balázs M., Kapás M., Ágai Csongor É., Domány G., Kiss B., Szombathelyi Z. (2008). Subnanomolar dopamine D3 receptor antagonism coupled to moderate D2 affinity results in favourable antipsychotic-like activity in rodent models: II. behavioural characterisation of RG-15.. Naunyn Schmiedebergs Arch. Pharmacol..

[19] Duric V., Banasr M., Franklin T., Lepack A., Adham N., Kiss B., Gyertyán I., Duman R.S. (2017). Cariprazine exhibits anxiolytic and dopamine D3 receptor-dependent antidepressant effects in the chronic stress model.. Int. J. Neuropsychopharmacol..

[20] Do A., Keramatian K., Schaffer A., Yatham L. (2021). Cariprazine in the treatment of bipolar disorder: Within and beyond clinical trials.. Front. Psychiatry.

[21] Murphy B.L., Babb S.M., Ravichandran C., Cohen B.M. (2014). Oral SAMe in persistent treatment-refractory bipolar depression: A double-blind, randomized clinical trial.. J. Clin. Psychopharmacol..

[22] Martinotti G., Dell’Osso B., Di Lorenzo G., Maina G., Bertolino A., Clerici M., Barlati S., Rosso G., Di Nicola M., Marcatili M., d’Andrea G., Cavallotto C., Chiappini S., De Filippis S., Nicolò G., De Fazio P., Andriola I., Zanardi R., Nucifora D., Di Mauro S., Bassetti R., Pettorruso M., McIntyre R.S., Sensi S.L., di Giannantonio M., Vita A. (2023). Treating bipolar depression with esketamine: Safety and effectiveness data from a naturalistic multicentric study on esketamine in bipolar *versus* unipolar treatment‐resistant depression.. Bipolar Disord..

[23] Heerlein K., Perugi G., Otte C., Frodl T., Degraeve G., Hagedoorn W., Oliveira-Maia A.J., Perez Sola V., Rathod S., Rosso G., Sierra P., Malynn S., Morrens J., Verrijcken C., Gonzalez B., Young A.H. (2021). Real-world evidence from a European cohort study of patients with treatment resistant depression: Treatment patterns and clinical outcomes.. J. Affect. Disord..

[24] Heerlein K, De Giorgi S, Degraeve G, Frodl T, Hagedoorn W, Oliveira-Maia AJ, Otte C, Perez Sola V, Rathod S, Rosso G, Sierra P, Vita A, Morrens J, Rive B, Mulhern H.S, Kambarov Y, Young AH (2022). Real-world evidence from a European cohort study of patients with treatment resistant depression: Healthcare resource utilization.. J. Affect. Disord..

[25] Crown W.H., Finkelstein S., Berndt E.R., Ling D., Poret A.W., Rush A.J., Russell J.M. (2002). The impact of treatment-resistant depression on health care utilization and costs.. J. Clin. Psychiatry.

[26] Fountoulakis K.N., Yatham L.N., Grunze H., Vieta E., Young A.H., Blier P., Tohen M., Kasper S., Moeller H.J. (2020). The CINP Guidelines on the definition and evidence-based interventions for treatment-resistant bipolar disorder.. Int. J. Neuropsychopharmacol..

[27] Diaz A.P., Fernandes B.S., Quevedo J., Sanches M., Soares J.C. (2022). Treatment-resistant bipolar depression: Concepts and challenges for novel interventions.. Br. J. Psychiatry.

[28] Malhi G.S., Bell E., Boyce P., Bassett D., Berk M., Bryant R., Gitlin M., Hamilton A., Hazell P., Hopwood M., Lyndon B., McIntyre R.S., Morris G., Mulder R., Porter R., Singh A.B., Yatham L.N., Young A., Murray G. (2020). The 2020 royal australian and new zealand college of psychiatrists clinical practice guidelines for mood disorders: Bipolar disorder summary.. Bipolar Disord..

[29] Blonde L., Khunti K., Harris S.B., Meizinger C., Skolnik N.S. (2018). Interpretation and impact of real-world clinical data for the practicing clinician.. Adv. Ther..

[30] Román V., Gyertyán I., Sághy K., Kiss B., Szombathelyi Z. (2013). Cariprazine (RGH-188), a D3-preferring dopamine D3/D2 receptor partial agonist antipsychotic candidate demonstrates anti-abuse potential in rats.. Psychopharmacology.

[31] Yatham LN, Vieta E, McIntyre RS, Jain R, Patel M, Earley W (2020). Broad efficacy of cariprazine on depressive symptoms in bipolar disorder and the clinical implications.. Prim. Care. Companion. CNS. Disord..

[32] Ragguett R.M., McIntyre R.S. (2019). Cariprazine for the treatment of bipolar depression: A review.. Expert Rev. Neurother..

[33] Joyce J.N., Millan M.J. (2005). Dopamine D3 receptor antagonists as therapeutic agents.. Drug Discov. Today.

[34] Citrome L., Yatham L.N., Patel M.D., Barabássy Á., Hankinson A., Earley W.R. (2021). Cariprazine and akathisia, restlessness, and extrapyramidal symptoms in patients with bipolar depression.. J. Affect. Disord..

[35] Earley W.R., Burgess M., Rekeda L., Hankinson A., McIntyre R.S., Suppes T., Calabrese J.R., Yatham L.N. (2020). A pooled post hoc analysis evaluating the safety and tolerability of cariprazine in bipolar depression.. J. Affect. Disord..

